# COVID-19 effects on operating room cancellations at a pediatric tertiary care hospital: A retrospective cohort study

**DOI:** 10.1016/j.amsu.2022.104427

**Published:** 2022-08-20

**Authors:** Wen Jiang, Daniela Carvalho

**Affiliations:** a Department of Otolaryngology, University of California San Diego, San Diego, CA, USA; b Rady Children's Hospital San Diego, San Diego, CA, USA

**Keywords:** Same-day surgery cancellation, COVID-19, Elective surgery, Pediatrics, Operating room

## Abstract

**Background:**

Same-day surgery cancellation results in decreased operating room (OR) utilization, reduced productivity, and inconvenience for patients. We aim to assess the cancellation rates of elective surgeries, identify common causes, and evaluate changes due to the COVID pandemic.

**Methods:**

A retrospective cohort study was conducted identifying all same-day surgery cancellations at a tertiary pediatric academic hospital from 1/1/2015 to 12/31/2017 (pre-COVID) and from 4/1/2020 to 3/31/2021 (post-COVID). Statistical analysis was performed using generalized regression with cancellation as the dependent variable. Period, age, ethnicity, gender, preferred language, and insurance were independent variables.

**Results:**

There were 55465 scheduled cases (41670 before and 13795 after COVID), with 1508 cancellations (2.7%). Of those, 1247 (3.0%) were before COVID and 261 (1.9%) after COVID (p < .001). Of all cases, 56.7% (31475) were male, 55.1% (30595) were non-Hispanic/Latinx whites, 82.3% (45638) spoke English, and 45.5% (25237) had public insurance. The mean age was 8.5 years (SD = 6.03).

The decrease in the probability of cancellation was most significant in patients with public insurance, < 1 year-of-age, Hispanic/Latinx who spoke Spanish (pre-pandemic = 4.9% [CI = 4.2%–5.8%]; pandemic = 2.8% [95% CI = 1.9%–4.0%]. Regardless of the period, Hispanic/Latinx patients and those with public insurance had higher rates of surgery cancellations (p < .001).

**Conclusion:**

There was a significant decrease in same-day cancellations post-pandemic. We hypothesize that the required pre-operative COVID test helped to minimize same-day cancellations. Increased communication and education enhanced family engagement and was critical for improved OR metrics, including cancellation rates.

**Level of evidence:**

level IV.

## 1 Introduction

Same-day operating room (OR) cancellation is a significant issue affecting hospitals world-wide [1,2]. Cancellations often waste valuable OR time causing decreased overall OR efficiency. It affects productivity of surgeons, anesthesiologists as well as OR nursing and support staff [3]. Cancellation and rescheduling also cause significant inconvenience with negative emotional implications for patients and families [4]. Most of the publications on this topic report rates for adult surgery cancellations between 4 and 14% [5]. In the pediatric population, viral respiratory illnesses and other medical conditions may make cancellation rates even higher.

The aim of this study is to assess the cancellation rates of elective surgeries performed at a tertiary pediatric academic hospital, identify common causes and evaluate changes brought on by the COVID-19 pandemic.

## 2 Methods

A retrospective cohort study was conducted examining all patients who underwent elective procedures as well as those who cancelled the elective procedures on the same-day of the surgery from 1/1/2015 to 12/31/2017 (pre-COVID) and from 4/1/2020 to 3/31/2021 (post-COVID) at Rady Children's hospital, a 511-bed free-standing academic pediatric tertiary care hospital in San Diego, California. This study was reviewed and approved by the institutional review board (IRB) of Rady Children's hospital and University of California, San Diego (study # 181752). Data extraction from the electronic medical record (EMR) system used reporting work bench tool embedded in the EMR without retaining individual protected health information. Eligible cases were defined as elective cases that were on the published OR schedule by the end of the previous business day (5pm) before surgery and included only cases where patients were coming from home (outpatient or same-day admission). Any inpatient, trauma, urgent or emergent add-on cases were excluded from the analysis. This project was initiated well before the onset of the COVID-19 pandemic in order to analyze OR efficiency. Therefore, we used “baseline” data from 1/1/2015 to 12/31/2017 as “pre-pandemic.” After the onset of the COVID-19 pandemic, we obtained approval with an amendment to the study through the IRB to include the data from 4/1/2020 to 3/31/2021 and designated this period as “post-pandemic.”

Statistical analysis was performed using JMP Version 14 (SAS Institute Inc, Cary, NC), with two-sided p-values less than 0.05 considered to be statistically significant. Age differences between pre- and post-pandemic as well as comparison of the total cases with cancelled cases were analyzed using two-sample t-tests. A generalized regression with cancellation as the dependent variable was created. Period, age, ethnicity, gender, preferred language, and insurance were independent variables. A prediction profiler was used to optimize the generalized regression model.

The study is complaint with the STROCSS 2021 criteria for reporting of cohort studies in surgery [6], and is registered with Research Registry with a unique identifying number 8015 which is accessible using the following link: https://www.researchregistry.com/browse-the-registry#home/registrationdetails/62ac04f37dc393001ef2e571/.

## 3 Results

There was a total of 55465 scheduled cases (41670 before and 13795 after COVID), with 1508 cancellations, an overall rate of 2.7%. The cancellation rate during the pre-pandemic period was 3.0% (1247 cases); and the cancellation rate was significantly lower post-pandemic at 1.9% (261 cases) (p < .001). Of all cases, 56.7% (31475) were male, 55.1% (30595) were non-Hispanic/Latinx whites, which is defined as Americans who identify themselves as “white” and are not of Hispanic or Latino heritage according to the United States Census Bureau [7]. Within the cohort, 82.3% (45638) spoke English, and 45.5% (25237) had public insurance. The median age was 7.5 years, and the mean age was 8.5 years (SD = 6.03).

During the pre-pandemic period, there was a total of 41670 cases with 3.0% cancellation rate (Table 1). There were 56.8% males and 43.2% females, and cancellation rates were not affected by gender. Hispanic patients made up 44.5% of this cohort and their cancellation rate was significantly higher at 3.8% when compared to the 2.6% for non-Hispanic patients. Most patients spoke English (81.8%), and language was not significantly associated with increased cancellation. More than half of the cohort had public sponsored insurance (51.1%) and cancellation rate was significantly higher when compared to those with private insurance. During the post-pandemic period, there was a total of 13795 cases, which was almost identical to the annual case load as the pre-pandemic period (13890). The cancellation rate was significantly lower at 1.9%. The demographic of the post-pandemic cohort was similar to the pre-pandemic period, with 56.7% males, 43.8% Hispanics, 83.8% English-speaking and 50.9% having public insurance.

**Table 1:** Patient characteristics and cancellation rates pre- and post-pandemic.

	Pre-Pandemic				Post-Pandemic				
	Total	Surgery	Cancelled	Rate (%)	Total	Surgery	Cancelled	Rate (%)	p
N	41670	40423	1247	3.0	13795	13534	261	1.9	<0.001
									
Male	23652	22952	700	3.0	7825	7682	143	1.9	0.85
Female	18018	17471	547	3.1	5970	5852	118	2.0	
									
Hispanic	18537	17866	671	3.6	6043	5914	129	2.2	<0.001
Non-Hisp	22907	22346	561	2.4	7688	7620	132	1.7	
Unknown	226	211	15	6.6	64	64	0	0.0	
									
English	34074	33107	967	2.9	11564	11361	203	1.8	0.07
Spanish	6456	6421	236	3.7	1881	1836	45	2.5	
Other	1140	895	44	4.9	350	337	13	3.9	
									
Public	21304	20765	882	4.2	7019	6844	175	2.6	<0.001
Private	20366	19658	365	1.9	6776	6690	86	1.3	
									
Mean Age (SD)	8.4 (6.0)	8.4 (6.0)	7.3 (6.1)		9.3 (6.2)	9.3 (6.2)	8.6 (6.3)		

Interestingly, the COVID pandemic has positively impacted the OR utilization with significantly lower rate of cancellation compared to our baseline. Regardless of the period, patients who had public insurance and those of Hispanic ethnicity had a higher rate of surgery cancellations (p < .001). By optimizing the factors in the generalized model profiler we found that the decrease in the probability of cancellation rate was most significant in patients who had public insurance, younger than 1 year-of-age, were Hispanic/Latinx and spoke Spanish (pre-pandemic = 4.9% [CI = 4.2%–5.8%]; pandemic = 2.8% [95% CI = 1.9%–4.0%].

The mean age of the pre-pandemic cohort was 8.4 (SD 6.0), and those with same-day cancellation were significantly younger with a mean age of 7.3 (SD 6.1) (p < .001). The mean age of the post-pandemic cohort was significantly older at 9.3 (SD 6.2) when compared to the pre-pandemic cohort (p < .001), and those with same-day cancellation had a slightly younger mean age of 8.6 (SD 6.3) which was not statistically significant (p = .07). During the pre-pandemic period, the average scheduled case length for cancelled surgery was 80 min (SD = 62). During the post-pandemic period, the average scheduled case length for cancelled surgery was 90 min (SD = 74). In surgery, estimates of the cost of OR time vary widely without well-established benchmarks. In an effort to understand costs of care, Childers et al. calculated the mean cost of OR time at $36 to $37 per minute, using financial data from California's short-term general and specialty hospitals in FY2014 [8]. With an average of 90 min case length, the gross average cost would be $3240 to $3330 per cancelled case assuming the OR time was not able to be filled. During the post-COVID period, with the reduction of cancellation rate from 3.0% to 1.9%, this translated to approximately 153 less cancelled cases during the 12-month period, which in term translated to $495,720 to $509,490 potential cost saving annually.

**Fig. 1:**
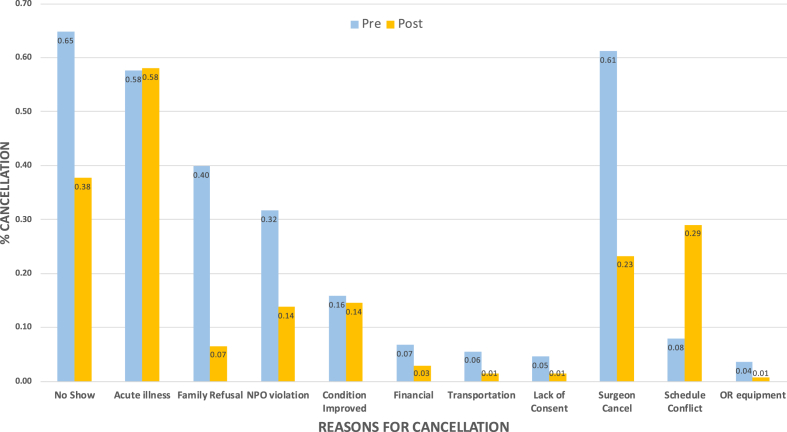
Reasons for cancellation, pre- and post-pandemic. Non-preventable patient-related factors, acute illness and condition improved both remained stable while all other preventable factors decreased. Cancellation rate due to scheduling conflict increased post-pandemic.

When analyzing reasons for cancellation, we separated the reasons into patient-related and non-patient-related categories (Fig. 1). During the pre-pandemic period, the most common patient-related reason was no-show with a cancellation rate of 0.65%, followed by acute illness (0.58%), patient/family refusal (0.40%), fasting or NPO violations (0.32%), condition improved (0.16%), financial (0.07%), transportation (0.06%) and inability to obtain consent (0.05%). The most common non-patient related reason for cancellation was because the surgeon deemed the procedure unnecessary (0.61%), followed by OR scheduling conflicts (0.08%) and OR equipment issues (0.04%). During the post-pandemic period, the most common patient-related reason was acute illness with a cancellation rate of 0.58%, followed by no show (0.38%), fasting or NPO violations (0.14%), condition improved (0.14%), patient/family refusal (0.07%), financial (0.03%), transportation (0.01%) and inability to obtain consent (0.01%). The most common non-patient related reason for cancellation was because OR scheduling conflicts (0.29%), followed by surgeon deemed the procedure unnecessary (0.23%), and OR equipment issues (0.01%). The rates of cancellation remained unchanged for the two patient-related reasons that are considered non-preventable: acute illness and condition improved; whereas all other categories decreased. The rate of cancellation due to OR scheduling conflict increased during the post-pandemic period.

## 4 Discussion

The same-day elective surgical case cancellation rate reported during baseline pre-pandemic period was quite low compared to many published reports [9], and this was reduced further after the onset of the COVID-19 pandemic. Unlike other reports of drastic decrease in elective surgical volumes [10], our institution experienced only a decrease in case load during the initial two months of the pandemic due to state mandated COVID-19 restrictions with a rapid subsequent return to baseline. With similar annual case load during the pre-pandemic and post-pandemic period, we feel that the comparison for cancellation rate was equitable. With the rough cost estimations, we have demonstrated that by reducing the rate of cancellation, there may be a significant improvement in overall OR efficiency with reduced cost and increased productivity.

We found that both Hispanic ethnicity and public insurance status were associated with increased probability of same-day cancellation. This likely reflects a degree of disparity in care, and private insurance has been found to be associated with decreased rate of cancellation even in countries with universal healthcare[11]. The other possibility is that these demographic factors may be an indirect reflection on other cultural, language or literacy barriers in communication between the healthcare providers and patients and their families.

After the onset of the COVID-19 pandemic, there was a significant change in pre-operative workflow regarding scheduling of elective surgical cases. At our institution, COVID-19 RT-PCR testing became a requirement very early on during the pandemic in order to keep the healthcare staff and patients safe. We stipulate that the mandatory pre-operative COVID testing required by the hospital 48 h prior to elective surgery helped minimize same-day cancellations. Although inconvenient for the family, the requirement of coming to the healthcare facility for the COVID test 2 days prior to surgery helped to reduce the “no show” rate since it is intuitive that if family already committed time and energy to come for the pre-operative testing, they are more likely to come on the day of the scheduled procedure. The extra visit could also allow the family to become acquainted pre-operatively with the facility (directions, parking) as well as allowing them to have another opportunity to clarify any pending questions before the procedure. This is reflected in the decreased cancellation rates related to the categories of “no show” and “family refusal.” Since the pre-operative COVID tests were all scheduled over the phone with verification of time and date of the surgery, the increased nursing communications [12] and education may have helped to reduce cancellations due to NPO violations.

It has been well-documented that since the onset of COVID-19, due to increased mask mandates and social distancing, there has been a significant reduction in respiratory illnesses in the pediatric population due to other viral pathogens [13]. At the onset of the study, we hypothesized that the decrease in viral respiratory illnesses among children especially in the younger age group may positively impact the cancellation rates. At the time of COVID testing, patients completed questionnaires regarding any respiratory symptoms such as fever, congestion, cough, etc. Many of these patients with positive respiratory symptoms were instructed to re-schedule the surgery rather than proceed with COVID tests. However, our analysis showed that the cancellation rate due to non-preventable patient-related causes such as “acute illnesses” and “condition improved” remained stable during the pre-pandemic and post-pandemic period with identical rate of cancellation due to acute illnesses. The reason behind this is unclear. We speculate that the acute illnesses may not be respiratory related illnesses or may be due to non-COVID viral illness that were not affected by masking mandates.

Not surprisingly, in the post-pandemic period, cancellation due to OR scheduling conflicts was significantly higher (0.29%) when compared to the pre-pandemic period (0.08%). This is likely due to periodic staffing shortage with “unscheduled time off” during COVID surges where the rates of the COVID-19 infection in the county were high, decreasing available OR block time.

The current study has a number of limitations. Given the retrospective nature of the study, documentation of the cancellation reasons may not always be accurate, and reasons may have been miss-classified. There may be other confounders that contributed to the cancellation rates that were not extracted or analyzed from the EMR. While many factors are significantly associated with increased risk of cancellation, the data presented here does not establish causality. Given our geographic location at the border between the United State and Mexico, the large proportion of Hispanic patients represented in our study mirrored the population composition of the San Diego county. Therefore, the demographic results reported here may not be generalizable to other geographic areas of the US. Given the variable mix of pediatric surgical subspecialties at individual children's hospitals, the reasons for cancellation due to both patient and surgeon-related causes may be different at another center since many of the reasons may be specialty dependent. Lastly, the data presented may not be easily extrapolated to other adult, non-tertiary, and international hospitals due to different surgical patient populations. However, the message that better patient education and communication are essential in improved care delivery is applicable in all healthcare settings.

The analysis gave us insights to the reasons for same-day cancellation. Although the overall rates reported here is lower compared to previously published data, we believe that there is still room for improvement as demonstrated by the decreased rate after the onset of the COVID-19 pandemic, especially in categories of patient-related reasons that are preventable while cancellation rate due to non-preventable reasons remained stable.

## 5 Conclusions

The COVID-19 pandemic has adversely impacted our entire healthcare system in the US since its onset in 2020. Although it has caused many inconveniences for both patients and providers, it did somehow have a positive impact in lowering our same-day elective surgical cancellation rate. We found that regardless of period, certain populations are vulnerable with increased risk of cancellation such has Hispanic ethnicity and public insurance status.

Once COVID infection rates decrease in the general population, we anticipate that pre-operative COVID testing may not be required for routine elective surgical procedures in the future. We would like to maintain the lower cancellation rate with the anticipated changes in workflow. Some of this may not be possible as we are already seeing increased rate of seasonal non-COVID viral upper respiratory infections especially in young children during 2021–22 season compared to the same period in 2020–2021 [11]. However, we believe that the increased communication with the families makes a positive impact on the same-day cancellation rates and we plan to continue the nursing phone call reminders close to the time of the scheduled surgery. We acknowledge that there may be significant challenges ahead in meeting this goal, especially with the current nursing and support staff shortages faced by all hospitals across the country. However, we have demonstrated that it is certainly possible to reduce cancellation rate even during the height of the COVID pandemic, and the potential cost-saving for the OR may allow us to develop future plans to incentivize increased pre-op nursing support. Bilingual nurses and increasing access to translators can also help with language barriers, and there is still more work to be done to assist families with low literacy levels (and other difficulties) in understanding and complying with pre-operative instructions.

## Ethical approval

This study was reviewed and approved by the institutional review board (IRB) of Rady Children's hospital and University of California, San Diego (study # 181752).

## Sources of funding

There is no funding source for this study.

## Author contribution

Study conception and design: Wen Jiang and Daniela Carvalho.

Data acquisition: Wen Jiang.

Analysis and data interpretation: Daniela Carvalho.

Drafting of the manuscript: Wen Jiang.

Critical revision: Daniela Carvalho.

## Consent

Not applicable for the current study.

## Registration of research studies

1. Name of the registry: researchregistry.com
2. Unique Identifying number or registration ID: researchregistry8015
3. Hyperlink to your specific registration (must be publicly accessible and will be checked): https://www.researchregistry.com/register-now#user-researchregistry/registerresearchdetails/62ac04f37dc393001ef2e571/

## Guarantor

None.

## Provenance and peer review

Not commissioned, externally peer-reviewed.

## Declaration of competing interest

None of the authors have anything to disclose.
